# Prediction of the Stability of the Casting Process by the HPDC Method on the Basis of Knowledge Obtained by Data Mining Techniques

**DOI:** 10.3390/ma17235935

**Published:** 2024-12-04

**Authors:** Marcin Brzeziński, Jakub Wiśniowski, Mariusz Łucarz, Karolina Kaczmarska, Alena Pribulová, Peter Futáš

**Affiliations:** 1 Faculty of Foundry Engineering, AGH University of Krakow, Reymonta 23 St., 30-059 Krakow, Poland; wisniowskij@agh.edu.pl (J.W.); eumar@agh.edu.pl (M.Ł.); karolina.kaczmarska@agh.edu.pl (K.K.); 2 Faculty of Materials, Technical University of Kosice, Metallurgy and Recycling, Letna 1/9, 042 00 Kosice, Slovakia; alena.pribulova@tuke.sk (A.P.); peter.futas@tuke.sk (P.F.)

**Keywords:** HPDC, porosity, aluminium alloys, quality, SPC

## Abstract

High-pressure die casting (HPDC) of aluminum alloys is one of the most efficient manufacturing methods, offering high repeatability and the ability to produce highly complex castings. The cast parts are characterized by good surface quality, high dimensional accuracy, and high tensile strength. Continuous technological advancements are driving the increase in part complexity and quality requirements. Numerous parameters impact the quality of a casting in the HPDC process. The most commonly controlled parameters include plunger velocity in the first and second phases, switching point, and intensification pressure. However, a key question arises: is there a parameter that can predict casting quality? This article presents an exploratory analysis of data recorded in a modern HPDC casting machine, focusing on the thickness of the biscuit. The biscuit is the first component of the casting runner system, with a diameter equivalent to that of the injection chamber and a height linked to various processes and mold characteristics. While its diameter is fixed, the thickness varies. The nominal thickness value and tolerances are defined by the process designer based on calculations. Although the thickness of the biscuit does not affect the casting geometry, it influences porosity and cold-shot formation. This study aimed to determine the relationship between biscuit thickness and casting quality parameters, such as porosity. For this purpose, a series of injections was produced using automated gating, and biscuit thicknesses were examined. This article presents quality assessment tools and statistical analyses demonstrating a strong correlation between biscuit thickness and casting quality. The knowledge gained from the methodology and analyses developed in this study can be applied in support systems for the quality diagnostics of HPDC castings.

## 1. Introduction

Casting is one of the most efficient methods for large-scale product manufacturing [1,2,3,4] and one of the least expensive technologies for producing metallic components or finished elements in a broad market [4,5]. Castings are often foundational parts of machinery and assemblies, and serve as a starting point across many industrial fields [3]. Compared to other production methods, casting offers numerous advantages, including minimized or even eliminated machining processes required for final products, the ability to produce parts with highly complex geometries that would otherwise require the assembly of multiple parts in alternative methods, and adaptability to mass production requirements [2,4,6]. The foundry industry is continuously advancing to meet the increasingly challenging demands of customers [2,7]. Additionally, given the extensive possibilities offered by a variety of metal alloys and their properties, specific branches of casting have distinct, specialized characteristics [8]. Consequently, both research institutions and companies are directing more financial resources toward the discovery of new alloys and specific casting methods, fostering new design solutions for equipment and manufacturing technologies to meet the stringent demands of various sectors, particularly the automotive industry, which is the most challenging one [2,6,8,9,10].

The automotive industry, one of the largest consumers of foundry products, is also the most demanding client [2,4,11]. Beyond technological, quality, and safety requirements, the industry imposes parameters related to repeatability and high production efficiency due to its mass-production nature [6]. Each year, the automotive sector produces millions of cast engine blocks, gearbox housings, and other automotive components. The industry shows growth trends in the coming years [12]. To meet such demands, the industry essentially mandates high-pressure die-casting (HPDC) technology, which is one of the most rapidly growing sectors of the casting industry [12]. Ensuring high productivity in this casting process requires controlling numerous factors, such as mold shape dimensions, die coating, chemical composition, and metal temperature, with particular attention paid to preventive actions to avoid potential defects [1,4,6,8]. HPDC castings are known for their high geometric precision, good mechanical properties, and low cost [11,13]. They are also widely used to produce electrical, hydraulic, and lighting components. Aluminum die casting offers numerous advantages over other metalworking processes, such as the ability to produce highly complex shapes that cannot be effectively achieved through extrusion or machining [13,14,15]. Additional benefits include the production of textured or smooth surfaces, casting of both large and small parts, and thin-walled components [16]. A prime example is the production of complex automotive parts, such as gearboxes and engine blocks [10].

The high injection speed of liquid metal and short mold filling time ensure that every area and hard-to-reach space in the mold is filled, precisely replicating the mold’s shape. These parameters enable the production of intricate castings, while a high intensification pressure improves the fill quality of the products [16,17]. Die-casting products meet stringent appearance and dimensional tolerance requirements [13] and are ideally suited for high-quality standards. Among the many benefits of die casting, one of the most significant is its exceptionally short casting cycle time. The short manufacturing time is especially well-suited for the mass production of complex, lightweight components [18,19]. High production efficiency allows for the rapid production of a large number of units, including both compliant and non-compliant parts, according to client specifications [2,13,20]. Minor undetected changes or incidents in the casting process can result in significant quality issues in large quantities [21]. Such instances significantly increase foundry costs related to sorting, scrapping, and implementing additional defect detection measures, making high repeatability the most desirable characteristic [4,6,9,21]. Both peripheral equipment and the casting machine, when operating correctly, have tolerances that generate “natural process variability” [9,22,23]. A major issue arises with variables that push equipment performance beyond this “natural variability” [21,24,25]. This necessitates detailed analyses and identification of the causes of disturbances, which require considerable effort, time, and data [22,25,26]. For high-volume production, preventive actions are essential [24]. Viewing casting as a set of parameters that must be met by the foundry, the process engineer should consider the casting process in a similar manner [27]. Casting represents the work of the entire team, and its inspection can confirm compliance with customer requirements [21,25]. Additionally, continuous process changes disrupt production stability, contrary to the need for repeatability [22,24,28]. To prevent this, the process designer should define the range of the monitored process parameters, establish their tolerances, develop control plans, and organize peripheral equipment inspections [28,29]. This approach enables an early response to emerging variability, allowing analytical actions to influence production quality while the process remains under control. Predicting potential changes in the production process allows for timely intervention in response to anomalies, thereby eliminating defective products and their causes [21,25,30].

However, like any manufacturing process, HPDC has drawbacks. The most important of these is the potential porosity due to air entrapment in the form of very fine bubbles, which affects the structural integrity of the casting and causes problems with subsequent mechanical and heat treatment. This problem is particularly relevant for components that must satisfy high-quality requirements, such as those in the aerospace and automotive industries. In addition, porosity limits heat treatment options, as gas trapped in the pores during heating can lead to deformation of the casting or the formation of defects. Another problem is the high start-up and operating costs of HPDC foundries. HPDC requires sophisticated equipment such as steel molds, casting machines, and cooling and lubrication systems. The cost of molds (dies) for HPDC is very high, making this process cost effective, mainly for mass production. In addition, HPDC machines are expensive to purchase and maintain. HPDC works best for metals and alloys with low melting points and good flowability, such as aluminum, magnesium, or zinc. Metals with high melting points, such as steel, are difficult to process in HPDC due to rapid mold wear and limitations in cooling technology. In addition, metals with low fluidity may not accurately fill the mold, resulting in underfilling and surface defects [1,8,13,20,26]. Porosity, primarily due to air entrapment during the filling phase, greatly affects casting quality [3,23,24,26,29,31,32]. HPDC technology involves injecting liquid metal under high pressure into a casting mold [19]. The injection chamber is filled with liquid metal manually—with an operator pouring a specified amount of metal using a ladle—or automatically—with a system dosing a precise amount into the chamber. The process then proceeds through three phases of plunger movement. The first movement occurs in the shot sleeve, where the alloy is forced forward by the plunger at a low speed of approximately 0.2–0.3 m·s^−1^ (depending on chamber filling and plunger diameter) until it nears the mold gate (for as long as the mold design allows for low-speed flow without disturbances, usually not beyond the gate) [24,32,33]. In the second movement, the alloy is transported at a high plunger speed of approximately 2–4 m·s^−1^, filling the casting and overflow/venting system to complete the mold filling. The third very short plunger movement at low speed occurs during the intensification stage and is aimed at compensating for shrinkage by pushing the last solidifying alloy under high pressure, typically between 20 and 100 MPa [8,18,22,31]. Additionally, during intensification, most of the entrapped air generated by the fluid flow in the shot chamber is compressed to relatively small sizes due to the applied pressure [31,34]. The presence of trapped air in the final porosity content is crucial for assessing the suitability of the cast component for quality and acceptance in the subsequent finishing stages [32,35]. Shrinkage porosity occurs in the final solidification phase, and its content depends on the design of the gating system and cavity-filling parameters. Voids in the casting structure arise from alloy shrinkage during solidification, gases present directly in the alloy, or a combination of these factors, including gas porosity from trapped air during high-pressure casting [31,32,34,36]. Unfortunately, complete elimination of porosity in HPDC castings is impossible [26]. Various techniques are employed to reduce it to acceptable levels, including increased third-phase pressure values, higher second-phase velocities, use of reduced pressure in the mold system, and optimization of the first (slow) phase to minimize entrapped air in the chamber and liquid metal-air contact [16,37]. Some of these methods may accelerate mold and plunger/cylinder mechanism wear and intensify unfavorable cavitation phenomena in the mold [18,19,29].

Another important factor affecting porosity in the finished die casting is mold design [27,28]. Designing the gating system, positioning individual castings, and venting are challenging and crucial, often preceded by extensive simulations verifying the design accuracy [27,33]. Key parameters guiding designers include rapid mold cavity filling with liquid metal, avoiding sharp angles that cause abrupt flow direction changes, and proper mold venting [16,17,27,31]. In addition to the design parameters, calculating the correct volume of metal needed to fill the mold cavity is critical. This is the total metal volume, including the vents, casting, and gating system. Depending on casting size, the metal-to-casting ratio ranges from 25 to about 80% [32,34]. In analyzing each mold system design, the first component is the biscuit, which directly contacts the plunger. It has a diameter matching that of the injection chamber and a height tied to various processes and mold characteristics. Its diameter is fixed; however, its thickness can vary. The nominal thickness and tolerances are defined by the process designer based on simulations and calculations [32,36,38]. Although biscuit thickness does not directly affect the geometry of the finished product, it impacts non-geometric characteristics, such as mold cavity fill quality and porosity [38].

This study analyzes the correlation between biscuit thickness and the quality of finished castings. The correlation can determine the effect of this parameter on predicting porosity in cast elements at a very early production stage. This parameter serves as a basis for implementing potential changes [21,30]. The casting presented in this study is a critical component of automotive safety systems. The analyses performed will expand knowledge regarding production stability, define the quality of the casting directly responsible for human safety, and contribute to minimizing casting scrap.

## 2. Materials and Methods

**Figure 1:**
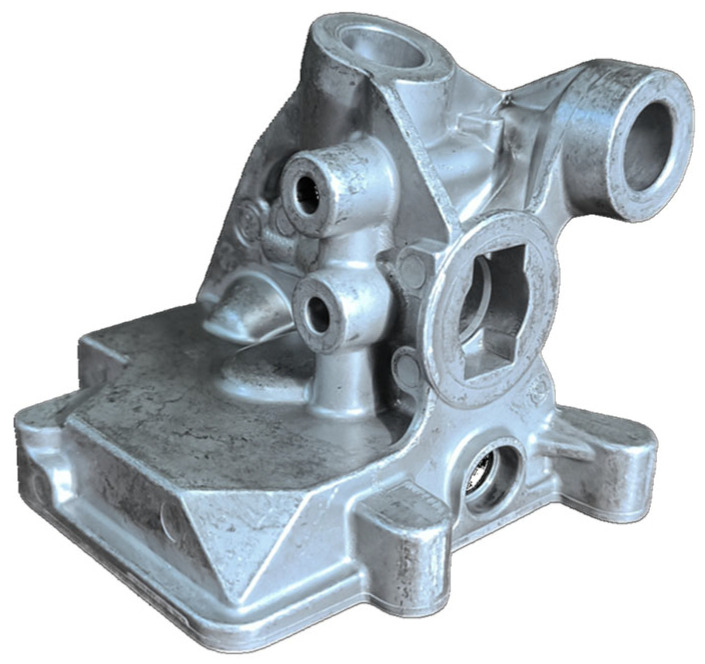
Tested casting.

**Figure 2:**
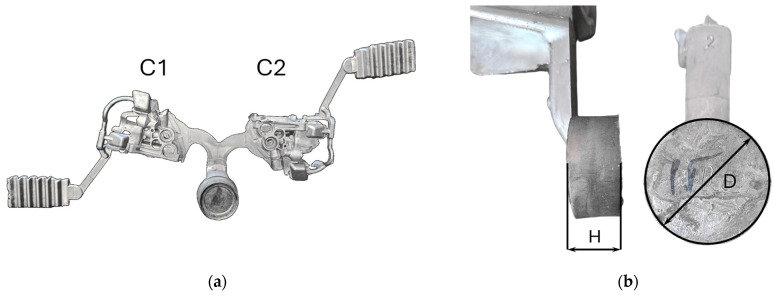
Casting biscuit: (**a**) die casting tree with two castings—socket C1 and C2, (**b**) HPDC biscuit—H: thickness, D: diameter.

The analysis and assessment of the impact of biscuit height on porosity, and consequently on the quality of the final product, were conducted using a casting (Figure 1) made with HPDC technology in a robotic production cell. The dimensions of the biscuit (Figure 2) were analyzed, focusing on their correlation with the porosity observed in the final castings.

The basic design parameters of the biscuit are presented in Table 1.

**Figure 3:**
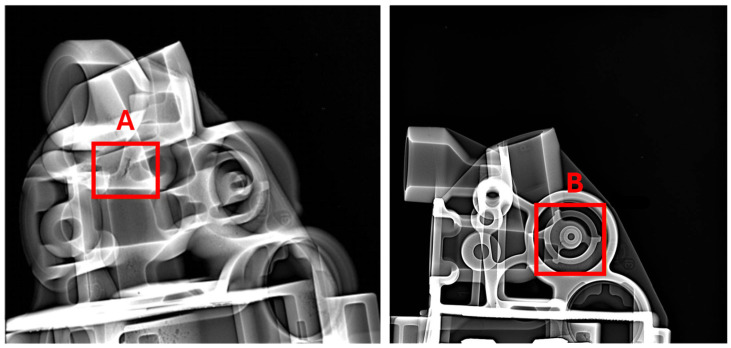
Porosity analysis areas (area A and area B—critical points).

Porosity evaluation in the castings was performed at two critical points (Area A and Area B), which are planned locations for CNC machining. These critical areas were examined using X-ray inspection, as illustrated in Figure 3.

**Figure 4:**
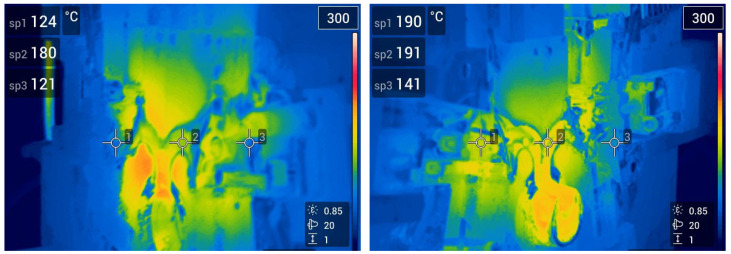
Thermal analysis of the casting mold.

To ensure consistency of results, the castings were produced using pressure die casting with fixed technological parameters throughout the cycle. Temperature measurements of the metal mold were conducted continuously to stabilize casting conditions. Relevant thermograms are shown in Figure 4. Information on the camera used and the survey methodology is provided in Section 2.3.

The gating system characteristics of the analyzed casting are presented in Table 2, and the technological parameters of the production process are summarized in Table 3.

### 2.1. Material

The base material was the EN AC-46000 AlSi9Cu3(Fe) casting alloy. The metal was melted in an industrial shaft furnace with a capacity of 3000 kg. The alloy composition was determined using a SPECTROMAXx SPECTRO emission spectrometer (SPECTRO POLAND, Kleve, Germany) on samples taken directly from the casting and in accordance with EN 1706:2020 + A1:2021 standards [39]. The tested composition is presented in Table 4. The metal was held in a Stotek Pro Dos 3 furnace (a pressure dosing system for supplying casting alloy to the machine and molds).

### 2.2. Test Casting and Process Parameters

**Figure 5:**
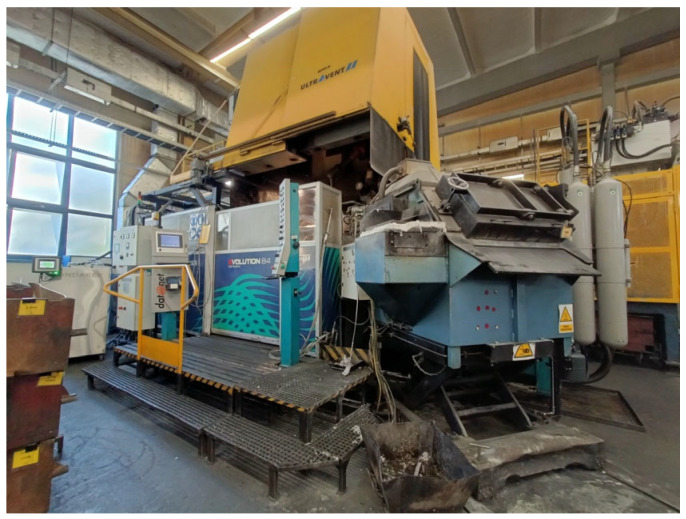
HPDC machine Buhler Evolutrion 84.

Foundries vary greatly in their degree of mechanization and automation. In HPDC, two methods of filling the pressure chamber are used: manual by the operator and fully automated. In the analyzed case, the casting machine was a modern automated die-casting machine—the Buhler Evolution 84 model. It is fully computerized, allowing for data collection and export to text files. The parameters of the pressure casting machine are provided in Table 5, and its appearance is shown in Figure 5.

The study was conducted on a robotic production cell comprising a casting machine, dosing furnace, robot for removing the casting along with the gating system, spraying robot, conveyor belt, and trimming press. The furnace places a specified amount of material into the shot chamber, followed by a shot, according to the technological settings. The data for the process are shown in Table 3. Next, the mold is halved separately, the ejector plate is activated, and the sprue is picked up by the removing robot. Meanwhile, the spraying robot applies a protective coating to the mold surface. The removing robot then transfers the sprue to the sensor area to confirm its completeness and places it on the conveyor belt, which transports the product to the operator. The worker places the casting in a trimming press to remove the residual air vent and gating system and visually inspects the trimmed casting. Based on the assessment, the casting is placed in either the OK or NOK bin.

**Figure 6:**
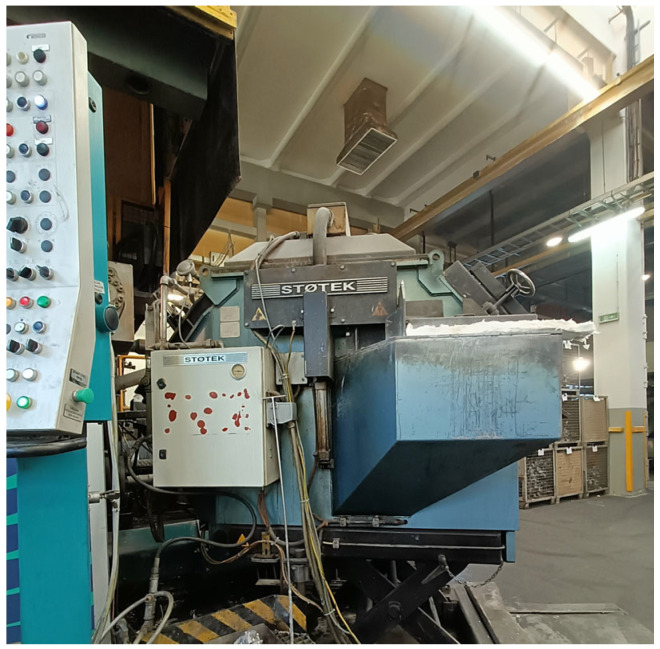
Stotek Pro Dos 3 pressure dispensing machine.

The Buhler chamber is supplied with metal by a pressure dosing system for casting machines and molds Stotek Pro Dos 3 (Figure 6). It is a resistance electric furnace that maintains the temperature for dosing aluminum alloys without a crucible. Filling the shot chamber with such devices involves air pressure exerted on the liquid metal. The dosing furnace is connected to the casting machine, where the dosing value is set in mass units—kilograms. The gas pressure increase exerts pressure on the liquid material, causing a pressure rise. To achieve pressure equilibrium, a flow is generated, moving the material towards the riser pipe. The liquid metal is then transferred to a heated transfer trough, flowing directly into the gating chamber. After this operation, the casting process proceeds according to the set technological parameters. The nominal biscuit thickness was 25 mm, with an allowable tolerance of +/−10 mm.

Automatic recording of biscuit thickness was conducted for 100% of the injections via the casting machine, and the data were exported to MS Excel. The number of measurement points: 4995. 

### 2.3. Methodology

**Figure 7:**
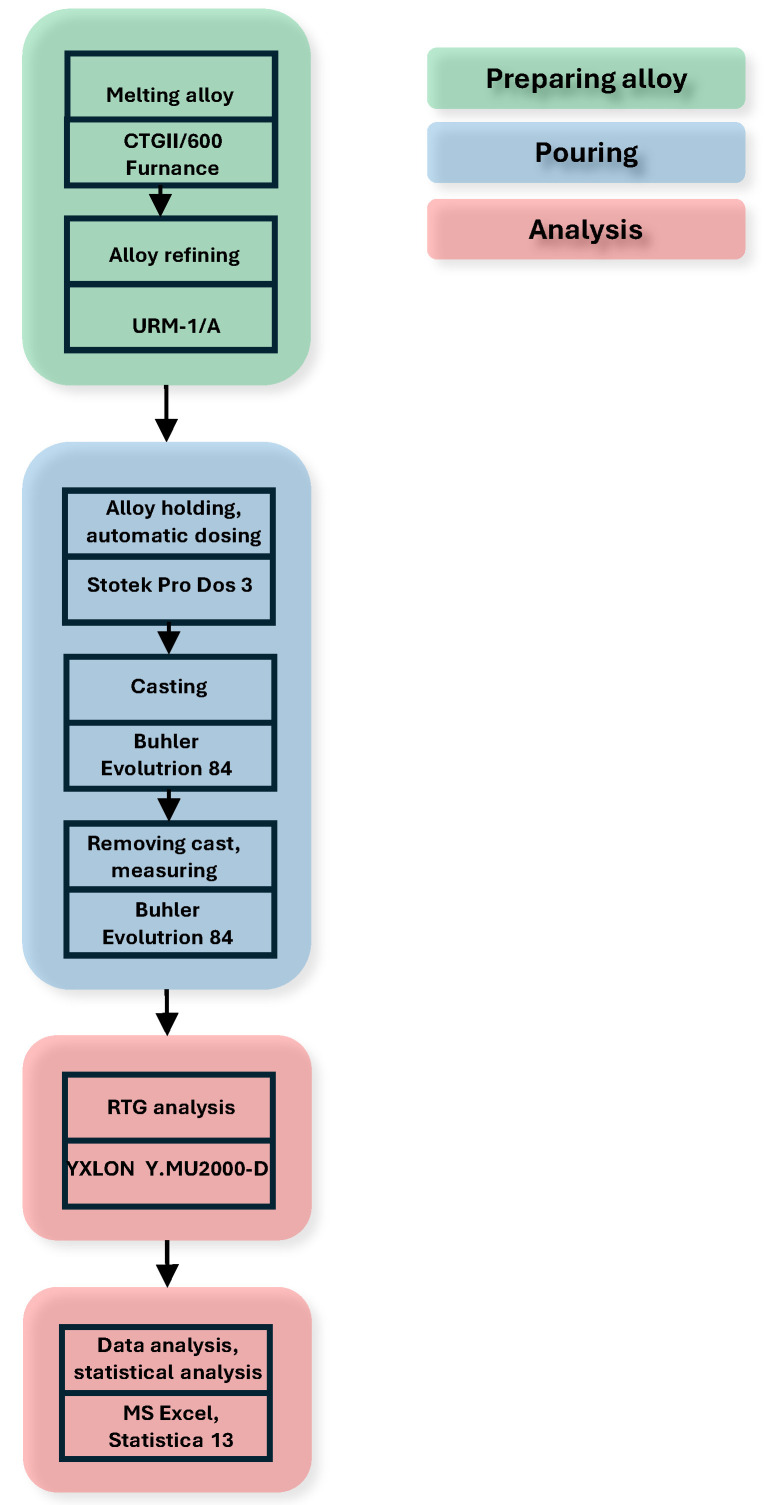
Diagram of the research methodology applied in the experiment.

The experimental methodology is presented in Figure 7.

**Figure 8:**
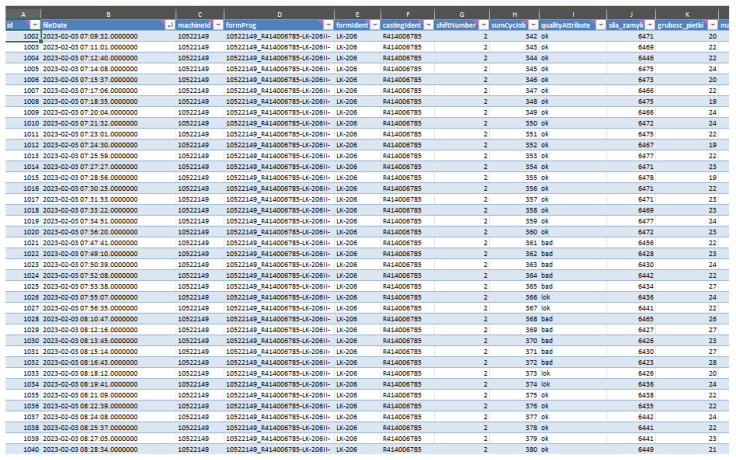
Sample data from Buhler Evolution 84.

The recorded data from the Buhler Evolution 84 machine were exported to a computer, and the results are shown in Figure 8.

**Figure 9:**
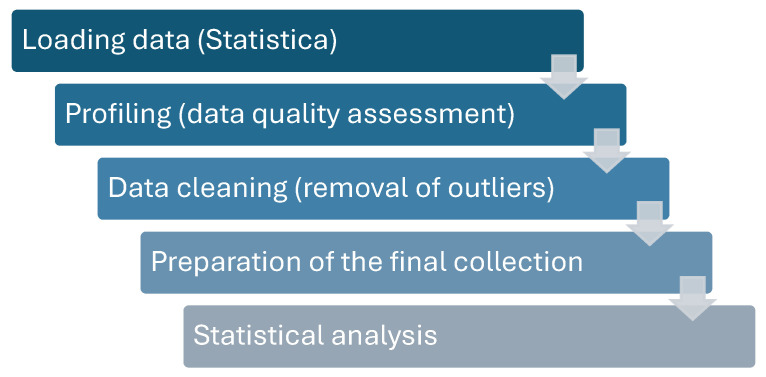
Diagram of data cleaning applied in the experiment.

The data were statistically analysed using Statistica 13.3 by StatSoft Europe GmbH. The data cleaning process was carried out entirely in Statistica. The methodology of data cleaning is presented in Figure 9. 

**Figure 10:**
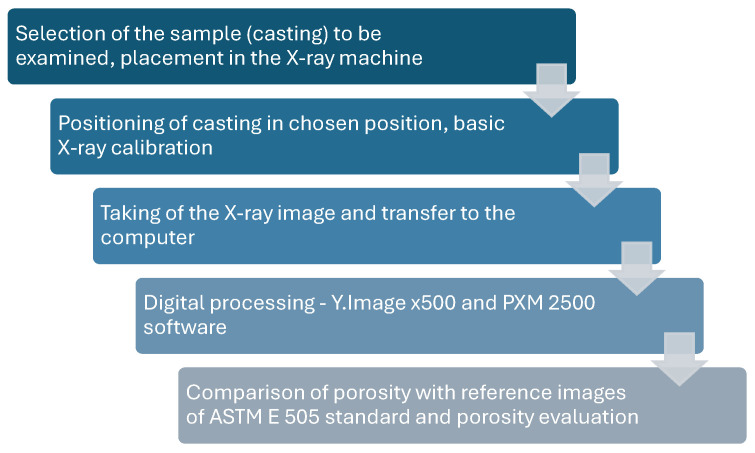
Schematic diagram of the X-ray research methodology used in the experiment.

The porosity of the castings was evaluated using a non-destructive method with an RTG machine—YXLON Y.MU2000-D. The castings were X-rayed, and the resulting images were assessed based on ASTM E 505—“Standard Reference Radiographs for Inspection of Aluminum and Magnesium Die Castings” and internal standards. The obtained results were used for qualitative evaluation of the castings. The detailed parameters of the machine are summarized in Table 6, while the casting analysis methodology is illustrated in Figure 10.

**Figure 11:**
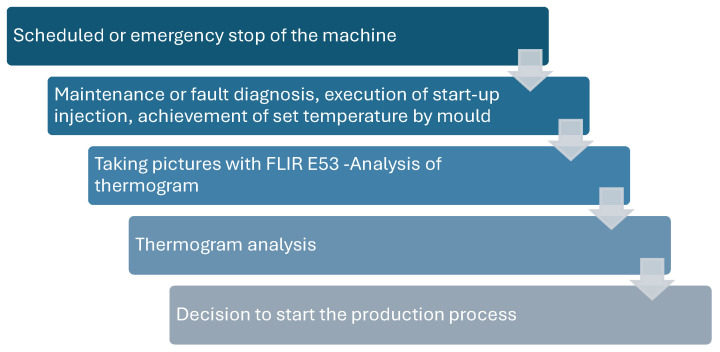
Schematic diagram of the thermal imaging methodology used in the experiment.

When the casting machine was stopped due to maintenance or breakdown, a thermal analysis of the mould was performed using a FLIR E53 from Teledyne Flir (Wilsonville, OR, USA). The Camera parameters are given in Table 7, and the methodology for performing the thermal analysis is shown in Figure 11.

## 3. Results

The obtained results are from the ongoing production of the foundry, and the data cover a 7-day production process. The analysis was divided into three parts. The first part includes statistical data analysis, descriptive statistics, data cleaning, normality distribution analysis, and time-course analysis. The second part is the RTG analysis, which determines the relationship between porosity and biscuit thickness. The third part contains an assessment of the dimensional stability of the biscuit.

### 3.1. Data Analysis

Given the large number of data points (4995 samples), preliminary statistical analysis was conducted to verify their accuracy. The analysis included basic descriptive statistics parameters, allowing the estimation of the distribution location, variability, and data structure. Measures such as the mean, median, standard deviation, variance, higher-order moments, and standard errors were used.

The mean, as a measure of central tendency, indicates the average biscuit thickness, while the median indicates the middle value, eliminating the impact of extreme values. The standard deviation and variance describe the dispersion level of the results around the mean, with the standard deviation being the square root of the variance, allowing for a data dispersion expression in the same units as the measurement.

Higher-order moments, such as skewness, measure the asymmetry of the distribution relative to the mean, identifying deviations towards higher or lower values. Kurtosis describes the degree of concentration of data around central values: higher kurtosis indicates more data clustered around the mean, reflected in a more peaked distribution curve and possibly indicating outliers.

The analysis also includes standard errors, assessing the precision of the mean and other parameters, indicating how representative the sample mean is for the entire population. The results of the statistical analysis are presented in Table 8.

Analysis of the data presented in Table 8 shows that both the mean and median biscuit thicknesses correspond to nominal values, suggesting central clustering of the results around the target. The measurements exhibit low variability, with athe coefficient of variation (CV) of 13%. The CV, defined as the ratio of the standard deviation to the mean, expresses data dispersion relatively independent of the measurement units.

The values of skewness and kurtosis confirm that the distribution of results is close to a normal distribution. Skewness close to zero indicates an even distribution around the mean, while kurtosis suggests a shape similar to a normal distribution, indicating the absence of extreme values.

However, extreme biscuit thickness values, from 10 mm (minimum value) to 40 mm (maximum value), indicate the presence of some anomalies and deviations from the desired biscuit thickness, suggesting the influence of random factors or process errors. As all the data contain a precise record of the time it was produced, it was possible to analyze why such values were being produced: the analysis showed that the outlier data came from injections made after the machine had stopped. In such cases, the machine carries out the process with special care for the mold, using reduced injection speeds and reduced compression pressure. These are the so-called start-up injections (castings from these injections are scrapped), which can deviate significantly from the standard tolerances. In the case analyzed, these were less than 15 mm or more than 35 mm (out of tolerance). Taking into account these data (whose values differ significantly from those assumed) could lead to situations in which the interpretation and conclusions drawn from the control cards could lead to incorrect decisions regarding the production process.

**Figure 12:**
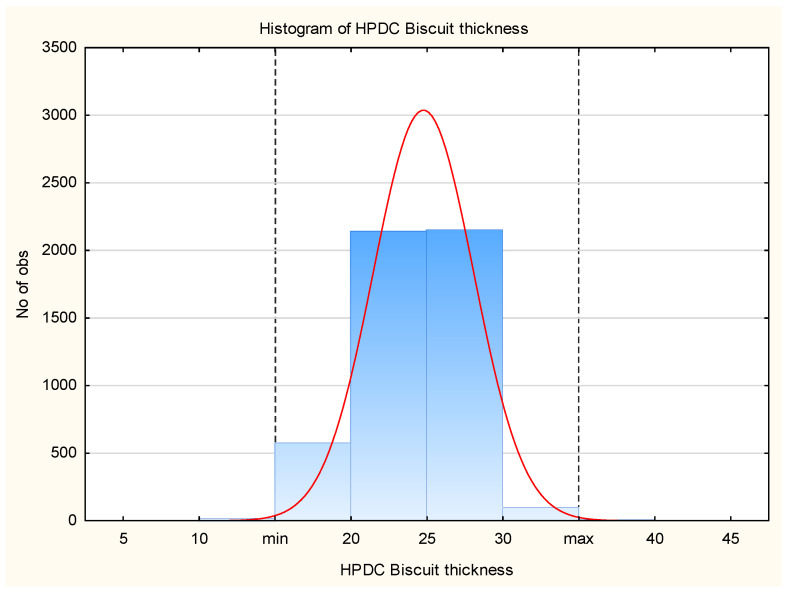
Histogram of HPDC biscuit thickness.

**Figure 13:**
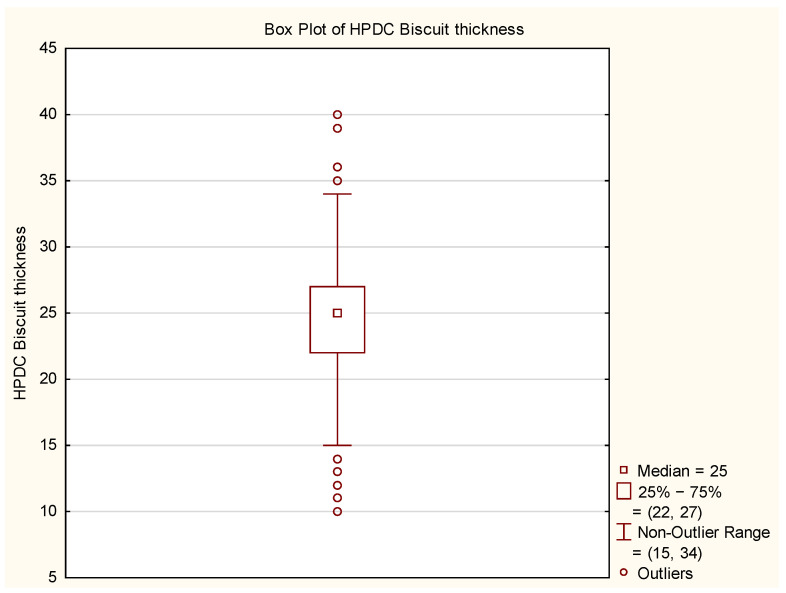
Box Plot of HPDC biscuit thickness.

For better data visualization, a histogram (Figure 12) and a box plot (Figure 13) were used. The histogram assesses the shape of the data distribution, while the box plot visually represents the range of variability, quartile positions, and outliers.

**Figure 14:**
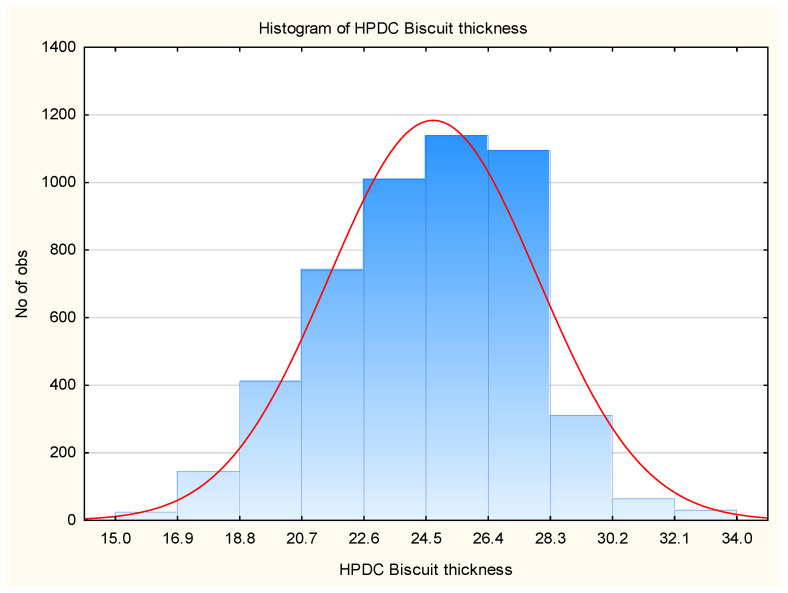
Histogram of HPDC biscuit thickness after data cleaning.

**Figure 15:**
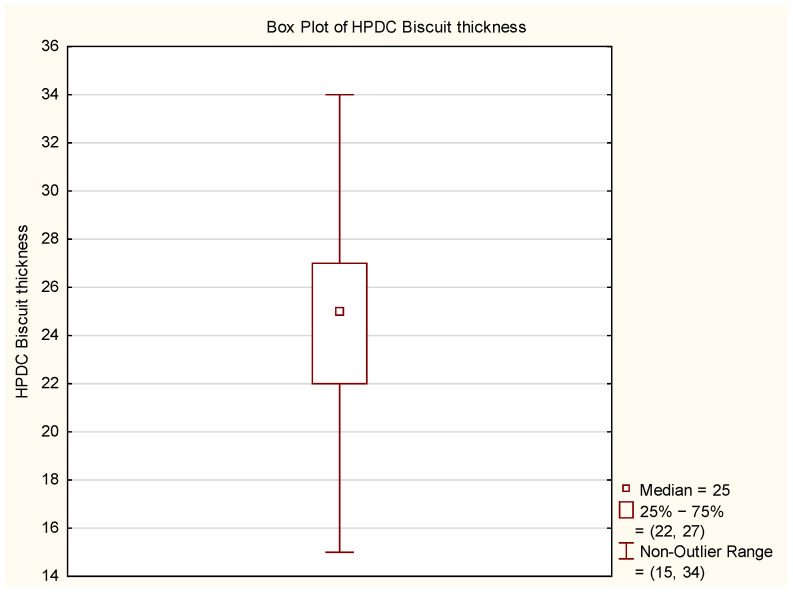
Box Plot of HPDC biscuit thickness after data cleaning.

Analysis of the obtained plots revealed outliers, whose origin needs to be analyzed concerning the source of such deviations. Additionally, the “T-shaped whiskers” are close to the maximum and minimum biscuit thickness values. Based on this information, the data were cleaned, and their graphical analysis is presented in Figure 14 and Figure 15.

After data cleaning, 25 measurements of biscuit thickness were excluded from further analysis, constituting approximately 0.5% of the total, which, from a statistical perspective, is negligible and does not significantly impact the results. Including them could cause significant measurement results, distortions, uncertainties in interpretation, leading to false conclusions and inappropriate decisions.

The next step in the analysis was to assess the changes in biscuit thickness during the production process. This analysis aims to reveal potential trends, fluctuations, and unforeseen changes over a long measurement period, which would not be visible using earlier tools.

**Figure 16:**
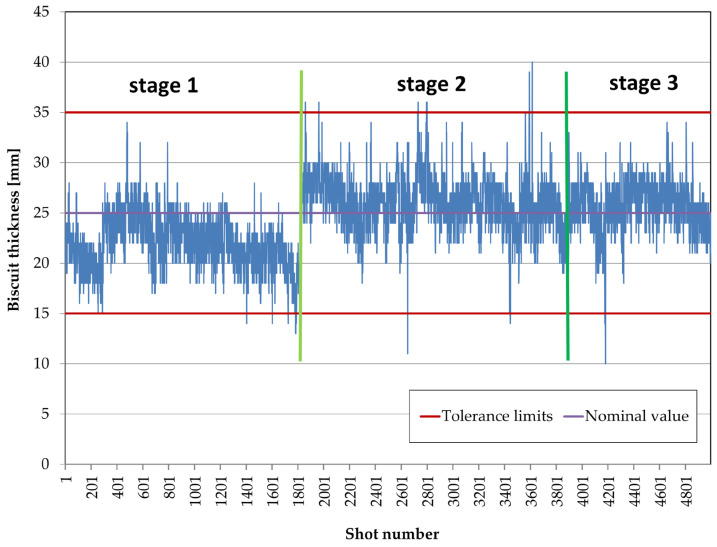
Box Plot of HPDC biscuit thickness after data cleaning.

Production processes often experience various failures that affect product parameters and sometimes cause machine stoppages. Figure 16 shows the thickness changes during the analysis period. Characteristic trends were identified, and their causes were verified using the tool shop department and machine monitoring records. Three characteristic stages were identified. At the end of stage 1, a machine stoppage occurred due to the need to clean a mold element and unblock material flow through the trough. This record indicates that the heated transfer trough transporting liquid metal was contaminated, reducing its flow capacity and affecting the amount of metal dosed. Any blockage in the trough resulted in less material in the shot chamber, lowering the biscuit thickness. At the end of stage 2, problems with the sealing of the mold’s movable element—the slider—were recorded. This led to a stoppage during which the tool shop department performed the regeneration work. After remounting and fitting the sliders, production continued. The effectiveness of the operation is confirmed by the absence of further sealing issues for the remainder of the measurement period.

### 3.2. Analysis of X-Ray Inspection

The castings underwent radiographic inspection to verify the porosity. Due to the volume of the produced components, it was not feasible to X-ray every product. Instead, representative samples were selected, considering production process variability and spanning a wide time range, to ensure that the results closely approximate what would be observed if every product had undergone radiographic inspection.

Porosity levels were evaluated according to ASTM E505 [35] guidelines and internal factory standards, with the adopted porosity classification detailed in Table 9.

**Figure 17:**
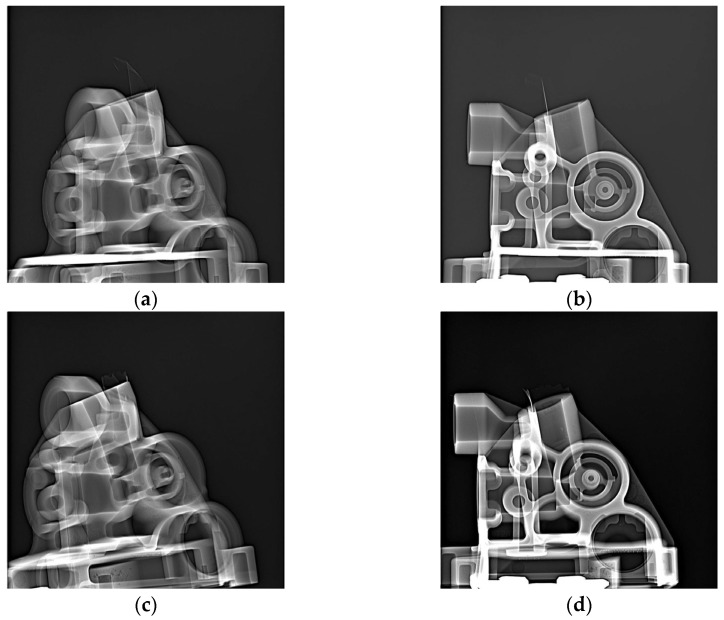
X-ray inspection for nominal (about 26 mm) value of biscuit thickness in specific sockets: socket C1: (**a**) area A—rate value 1, (**b**) area B—rate value 2; socket C2: (**c**) area A—rate value 2, (**d**) area B—rate value 2.

**Figure 18:**
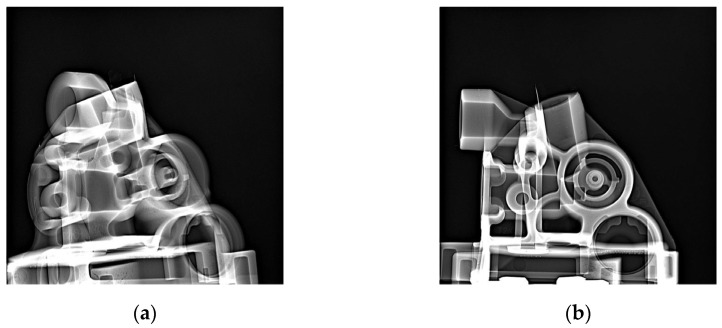

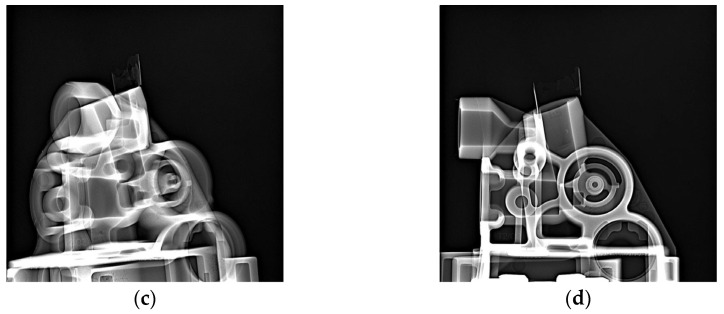
X-ray inspection for maximum (about 35 mm) value of biscuit thickness in specific sockets: socket C1: (**a**) area A—rate value 3, (**b**) area B—rate value 2; socket C2: (**c**) area A—rate value 3, (**d**) area B—rate value 2.

**Figure 19:**
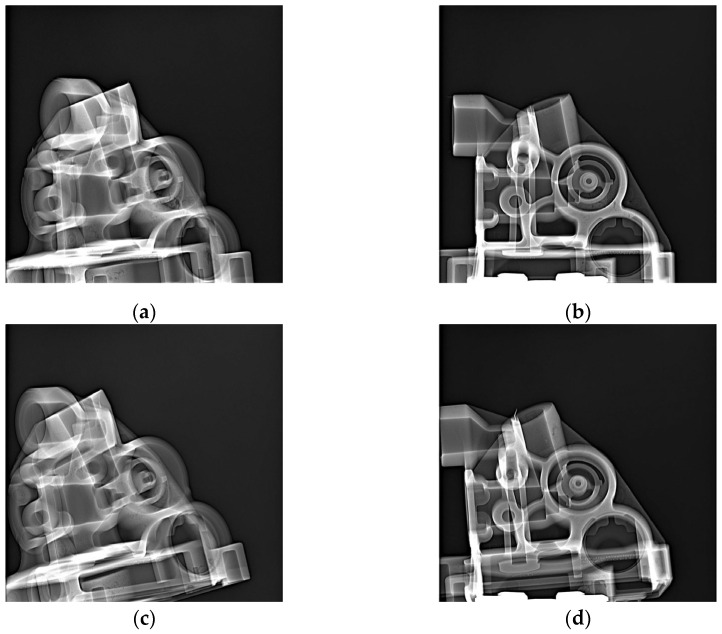
X-ray inspection for minimum (about 10 mm) value of biscuit thickness in specific sockets: socket C1: (**a**) area A—rate value 3, (**b**) area B—rate value 3; socket C2: (**c**) area A—rate value 3, (**d**) area B—rate value 3.

Figure 17, Figure 18 and Figure 19 show example X-ray images for three thicknesses of the biscuit as per tolerance criteria. Figure 17 depicts the thickness within the tolerance, Figure 18 shows the thickness above the upper tolerance, and Figure 19 shows the thickness below the lower tolerance. Two critical areas (A and B) (marked in Figure 3) were highlighted regarding further processing, along with two sockets (Socket C1 and C2) related to the casting tree structure.

**Figure 20:**
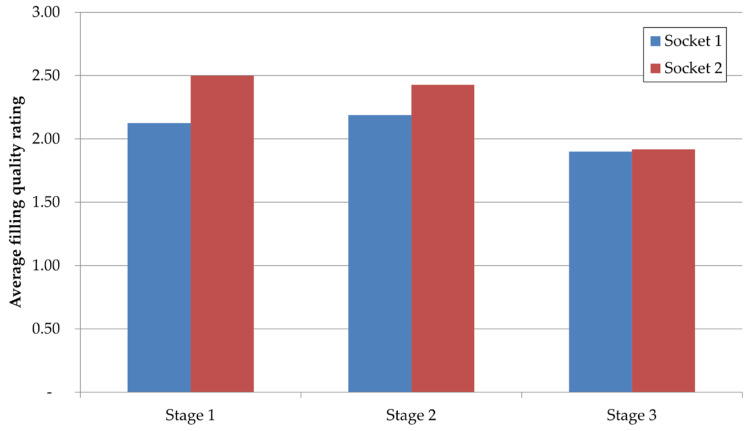
Average quality ratings based on X-ray images.

**Figure 21:**
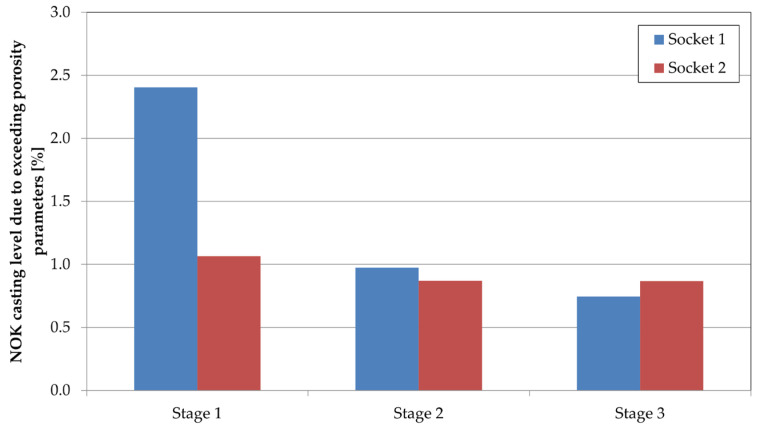
Defective castings due to porosity issues on machining surfaces.

The analysis in Figure 16 enabled the segmenting of the process into phases for qualitative evaluation of casting quality regarding defect levels. This analysis utilized radiographic (X-ray) inspection data. Figure 20 and Figure 21 present these results. Figure 20 shows the average rejection rate (in ASTM classification) at each stage, while Figure 21 indicates defect levels due to porosity inconsistencies on surfaces designated for machining.

Based on Figure 20, phases 1 and 2 exhibit similar cumulative quality levels according to X-ray assessment, reaching around 2.31/3. The third, final phase shows a decrease to around 1.9/3, marking an improvement of approximately 18%. This rejection rate reduction resulted from efforts to seal the movable mold part—the slider—ensuring that previously leaking material stayed within the mold, improving material feed for the part.

Figure 21 presents a percentage analysis of castings failing to meet porosity requirements. Phase one had a relatively high rejection rate for porosity on machined surfaces (the average value for sockets was 1.74%). This level decreased across subsequent phases, from 0.92% to 0.79%, marking a 55% reduction from the initial value. This improvement was achieved by stabilizing the process and adjusting the biscuit thickness closer to the nominal value.

### 3.3. Dimensional Stability Assessment of Biscuit Thickness

A preliminary statistical analysis of the biscuit thickness measurements identified three production process phases. An XmR (Shewhart’s Control Chart; individuals and moving range) control chart was chosen to assess biscuit thickness stability, enabling variability monitoring and identifying the causes of fluctuations. Control chart analysis involves plotting a selected statistic for a quality characteristic by examining values against control lines over time [40].

Given the identified process phases, separate control charts are recommended for each, allowing precise stability assessments for each phase. An XmR control chart was chosen due to the data characteristics (individual measurements).

**Figure 22:**
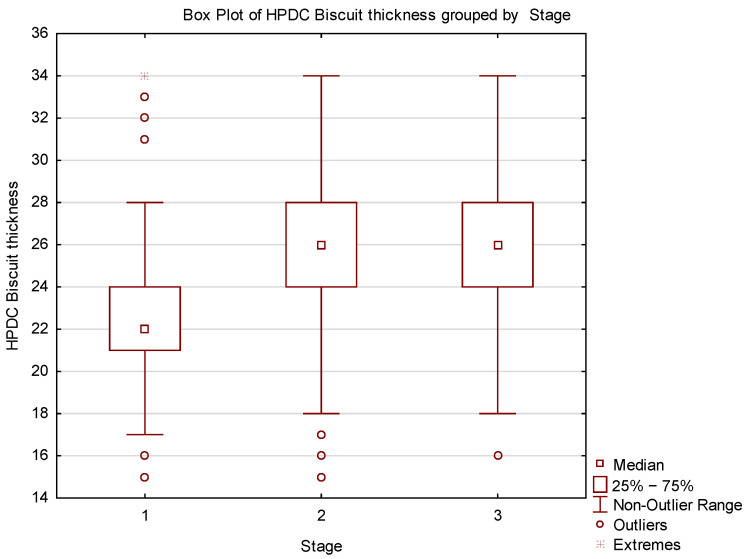
Box Plot of biscuits grouped by stage.

Figure 22 uses a box-and-whisker plot to analyze the variability in each phase, showing a gradual stabilization of biscuit thickness. Phase one displays significant outliers, indicating substantial thickness dispersion caused by clogged transport channels and metal feed variability. Phase two shows greater stability, although a new source of variability—mold sealing issues—arose. After correcting this issue, the process achieved its highest stability.

**Figure 23:**
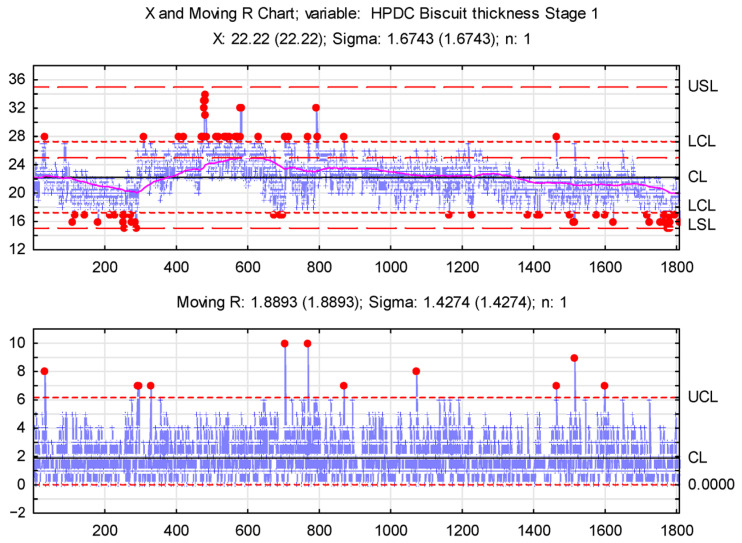
X and Moving R Chart—stage 1.

**Figure 24:**
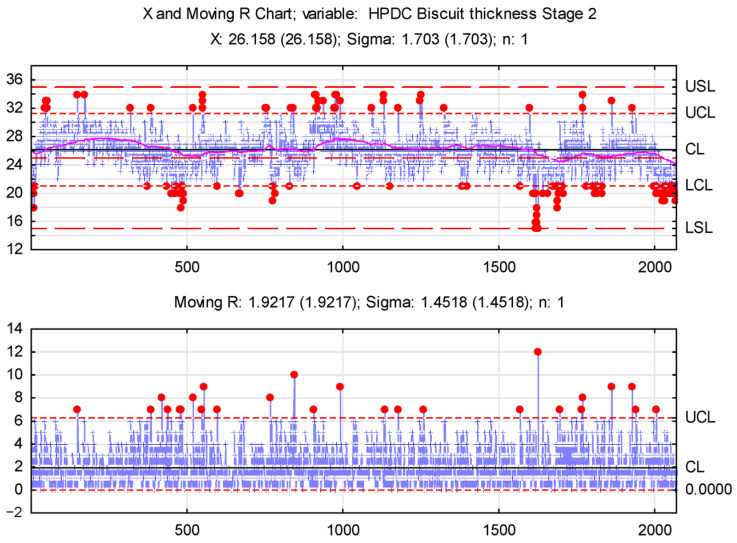
X and Moving R Chart—stage 2.

**Figure 25:**
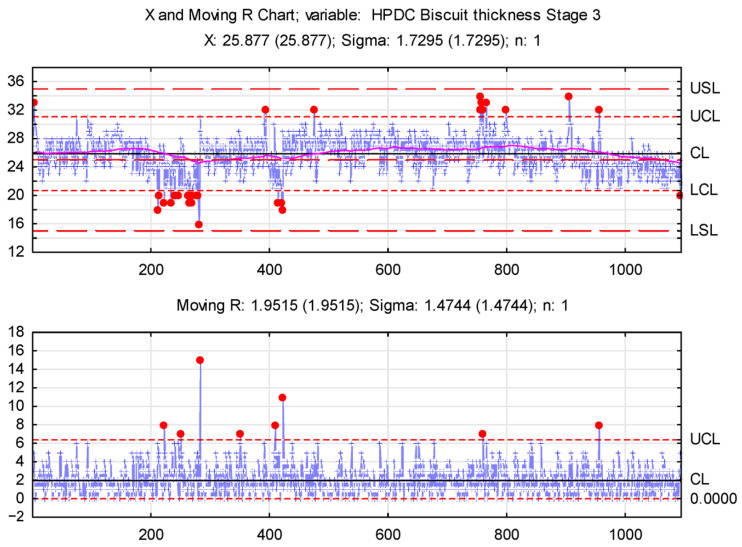
X and Moving R Chart—stage 3.

XmR control charts are shown in Figure 23, Figure 24 and Figure 25.

The data in Figure 23 reveal high biscuit thickness variability, especially during the initial production phase. Both lower (LCL) and upper control limits (UCL) were breached, with individual measurements nearing USL and LSL (Upper/Lower Specification Limits). Notably, values below the LCL could increase the porosity of the castings. The X-chart’s center line (CL) shows a significant gap between the mean thickness and the nominal value, resulting from contaminants in the pouring channel. This problem escalated, prompting operator intervention and a process halt to clear the channel.

Subsequent stages saw process stabilization. A moving average curve on the chart allows for long-term trend observation, revealing a gradual decrease in biscuit thickness due to channel clogging.

Figure 24 illustrates the production process after unclogging the transport pipe. The moving average analysis indicates stabilization without a clear trend; however, biscuit thickness variations exceeding both nominal and undersized values require further investigation. These fluctuations likely resulted from intermittent pouring channel cleaning by the operator, which was performed without stopping the machine. Toward the end of this stage, a concerning downward trend and increased thin biscuits were noted, attributed to a leak in the movable mold element—the slider.

Loss of mold sealing causes flash, the thickness and spread of which depend on the leak severity. The molten metal intended for the part flows into the mold seams, creating a flash that hinders casting and may cause dimensional nonconformities. Significant flash along mold seams reduces cavity fill, requiring the piston to travel further during dosing, thus reducing the biscuit thickness. The observed trends suggest that reduced mold sealing is directly correlated with decreased biscuit thickness during repeated dosing.

The third stage began once the mold sealing was restored. Maintenance records indicate occasional issues stemming from furnace malfunctions (heater element replacement and channel cleaning) (Figure 25). Control charts reflect significant production parameter stabilization, affirming that corrective actions achieved the desired outcome. However, the downward trend in the final phase requires further analysis to prevent future deviations.

The causes of the intervention are the subject of ongoing analysis. The phenomenon of trough clogging is mainly due to the direct contact between the liquid metal and air, resulting in the formation of oxides that block the flow in the trough. This results in a reduction in metal throughput and increased variability in biscuit thickness. As part of the optimization efforts, projects are being implemented to minimize the contact area between the molten metal and the air. In addition, the problem of mold sealing and leakage can be a consequence of suboptimal mold design, which is also an area of ongoing research and development. The result of this work is, among other things, the identification of the causes of their occurrence.

Gluing of the casting mold is most often the result of a reproducing component that is too hot. The causes of this problem may include the following:

- lack of a cooling system (design error),
- ineffective cooling resulting from
  ✓ insufficient cooling range,
  ✓ inadequate design of cooling channels (e.g., too small diameter),
  ✓ use of unsuitable cooling medium,
  ✓ clogging of existing cooling channels during operation.

In the case of mold leaks, e.g., on sliders, the problem is not always related to insufficient mold short-circuiting. Possible causes are:

- too high a post-pressure or too high a pressure in the mold cavity,
- wrong slide fit,
- insufficient force generated by the slider actuators,
- too high a melt temperature,
- too early, a switch from phase II to phase III injection.

The above problems can be taken into account when designing a new tool to improve the performance of the mould and increase its productivity and service life.

## 4. Discussion

The conducted studies align with the existing literature, demonstrating that biscuit thickness—and consequently the volume of poured metal—significantly affects casting porosity. Each analysis confirmed that reducing biscuit thickness increases casting porosity. This study aimed not only to describe the impact of biscuit height on porosity but also to analyze the correlation between biscuit thickness and porosity, enabling quality improvement by predicting porosity levels based on control chart trends for biscuit thickness. A standard control chart from the SPC system was employed to monitor process stability, allowing early detection and response to deviations. Dividing the process into phases (considering incidents) permitted a more detailed statistical analysis for each phase. However, whole-process analysis shows that the results align with phase-based analyses, including those based on X-ray images. Both types of analyses complement each other, providing comprehensive tools for continuous quality improvement in foundry production and defect reduction. Reducing the variability of biscuit thickness is very important for a company’s quality policy. This phenomenon is related to the fact that greater variability in process parameters, such as material dosage, as seen, for example, in the variation of biscuit thickness, leads to an increased number of non-conforming products. A mold is characterized by a certain service life, expressed, for example, as 100,000 injection cycles, which translates into 200,000 castings. With low variability in biscuit thickness, lower scrap rates can be expected, allowing more full-value parts to be produced and sold from a single mold. However, the high variability in process parameters results in more waste (scrap), which reduces the number of saleable parts. Process stability, measured by controlling biscuit thickness variability, therefore, has a direct impact on mold utilization efficiency, reducing production costs, and increasing profitability.

## 5. Conclusions

In summary, the study findings lead to the following conclusions.

1. The high-pressure casting under study has high technological complexity and is prone to porosity in critical areas, affecting the quality and CNC processing outcomes.
2. X-ray analyses confirm a strong porosity dependence on the biscuit height, especially when deviating from the design specifications.
3. Precise dosing of molten metal into the shot chamber is crucial for reducing porosity.
4. Control charts enable porosity prediction in the final castings, thereby enhancing foundry production quality.
5. Control charts also allow the early detection of process incidents affecting porosity, supporting high product quality.
6. While automatic pouring ensures high repeatability, it does not eliminate the risk of tolerance deviations, necessitating process safeguards.
7. Dosing fluctuations lead to variable chamber fill, potentially resulting in suboptimal metal volume despite meeting the technological requirements, which may cause air occlusion in the shot chamber.
8. Ongoing adjustments to technological parameters must align with the current state of the pressure die-casting machine to optimize the production process.

## Figures and Tables

**Table 1:** HPDC biscuit parameters for the analyzed casting.

Parameters of HPDC Biscuit	
Property	Value
Biscuit height, mm	25 (+/−10)
Biscuit diameter, mm	70
Biscuit volume, cm^3^	96.21 (+/−38.48)

**Table 2:** Characteristics of the gating system.

Shot Chamber Properties	
Property	Value
Plunger diameter, mm	70
Active chamber length, mm	420
End of the filling hole, mm	70
Compression surface, cm^2^	310
Cast volume, cm^3^	83.33
Gating system volume, cm^3^	266.67
Overflow volume, cm^3^	55.56
Branch volume, cm^3^	488.89
Dosing volume, cm^3^	550

**Table 3:** Technological parameters of casting cycle.

Technological Parameters of the Casting Cycle	
Property	Value
Alloy	EN-AC 46000
Pouring temperature, °C	690
Die temperature, °C	190
Speed at the filler slot, m/s	53.3
Intensification pressure, MPa	83
Maximum pressure in the dosing furnace, kPa	7

**Table 4:** Chemical composition of the applied alloy.

Element	Value, %
Al	86.1
Si	8.8
Fe	0.87
Cu	0.87
Mn	0.195
Mg	0.15
Cr	0.044
Ni	0.08
Zn	1.03
Ti	0.051
Pb	0.084
Sn	0.021

**Table 5:** Technical parameters of Buhler Evolution 84.

Parameter	Specification
Type	Die casting machine—metal injection
Model	Buhler Evolution 84
Maximum casting weight	5.03 kg (Al)
Clamping force	850 tonnes
Machine weight	36.5 tonnes

**Table 6:** Technical parameters of X-Ray Machine YXLON Y.MU2000-D.

Parameter	Specification
Manufacturer	Comet Yxlon, Hamburg, Germany
Type	X-Ray Machine
Model	YXLON MU2000-D Standard
RTG Lamp	Y.TU 160-D06 160 kV
Rotary table	600 mm
Software including ASTM window	Y.IMAGE 2500-D
Frequency of basic calibration	before each test
Main frequency calibration	annually

**Table 7:** Technical parameters of FLIR E53.

Parameter	Specification
Type	Thermal Imaging Camera
Model	FLIR E53
Features E53 Thermal Sensitivity/NETD	<0.03 °C @ 30 °C (86 °F), 42° lens
IR Resolution	240 × 180 (43,200 pixels)
Object Temperature Range	0 °C to 650 °C
Image Frequency	30 Hz

**Table 8:** Statistical data analysis results.

Parameter	Specification
Valid N	4995
Percent of Valid Observation	100
Mean	24.6580581
Median	25
Minimum	10
Maximum	40
Variance	10.7577019
Standard deviation	3.27989358
Coefficient of Variation	13.301508
Standard Error	0.0464079096
Skewness	−0.189708083
Kurtosis	0.300340094

**Table 9:** Detailed classification of adopted porosity.

X-Ray Inspection Criteria	
Rate	Value
Fine porosity	1
Numerous porosities and small, isolated shrinkages	2
Increased porosity and large shrinkages	3

## Data Availability

The original contributions presented in this study are included in the article. Further inquiries can be directed to the corresponding author.
